# The Use of Electrochemical Methods to Determine the Effect of Nitrides of Alloying Elements on the Electrochemical Properties of Titanium β-Alloys

**DOI:** 10.3390/ijms24021656

**Published:** 2023-01-14

**Authors:** Jitřenka Jírů, Vojtěch Hybášek, Petr Vlčák, Jaroslav Fojt

**Affiliations:** 1 Department of Metals and Corrosion Engineering, University of Chemistry and Technology, Technická 5, 16628 Prague, Czech Republic; 2 Department of Physics, Faculty of Mechanical Engineering, Czech Technical University in Prague, Technicka 4, 16607 Prague, Czech Republic

**Keywords:** nitrogen ion implantation, titanium alloy, nitrides, corrosion

## Abstract

Titanium beta alloys represent the new generation of materials for the manufacturing of joint implants. Their Young’s modulus is lower and thus closer to the bone tissue compared to commonly used alloys. The surface tribological properties of these materials should be improved by ion implantation. The influence of this surface treatment on corrosion behaviour is unknown. The surface of Ti-36Nb-6Ta, Ti-36Nb-4Zr, and Ti-39Nb titanium β-alloys was modified using nitrogen ion implantation. X-ray photoelectron spectroscopy was used for surface analysis, which showed the presence of titanium, niobium, and tantalum nitrides in the treated samples and the elimination of less stable oxides. Electrochemical methods, electrochemical impedance spectra, polarisation resistance, and Mott–Schottky plot were measured in a physiological saline solution. The results of the measurements showed that ion implantation does not have a significant negative effect on the corrosion behaviour of the material. The best results of the alloys investigated were achieved by the Ti-36Nb-6Ta alloy. The combination of niobium and tantalum nitrides had a positive effect on the corrosion resistance of this alloy. After surface treatment, the polarization resistance of this alloy increased, 2.3 × 10^6^ Ω·cm^2^, demonstrating higher corrosion resistance of the alloy. These results were also supported by the results of electrochemical impedance spectroscopy.

## 1. Introduction

Titanium exhibits excellent corrosion resistance due to its oxide layer [1,2,3]. The resistance of the metal and its alloys is therefore directly dependent on the state of the protective barrier. One common problem is the repeated mechanical abrasion of the layer—fretting. Such a damaged surface can be corrosively attacked. One of the ways to increase the abrasion resistance and thus the corrosion resistance is to modify the surface layer. Thermal and anodic oxidation and ion implantation are the main subjects of research [3,4,5,6,7,8,9,10].

The most important advantage of ion implantation is that it is a modification of the existing surface, not a coating. This effect is used not only for the surface treatment of metals but also in the production of semiconductor components. This allows the implant to preserve its original elasticity and strength and have a surface with better wear resistance than the native surface. Titanium, especially β-alloys, has properties, such as elastic modulus, similar to human bone, which makes it very useful in the medical field. As stated before, there is concern regarding the wear resistance of the oxide layer. This is where nitrogen ion implantation can be helpful [5,7,8,11,12,13,14,15,16,17].

Ion implantation is performed under high vacuum and at low temperatures. In this process, the ions of an implanted element, nitrogen, for example, are accelerated and directed towards the modified material. Depending on the required implant depth, the acceleration voltage is set in the range of kV to several MV. In the first step, accelerated ions are formed from atoms with the help of electrons. These are then attracted to the oppositely acquired sample, passing through a magnetic field, where ions with the required parameters are selected. Furthermore, a series of electrostatic and magnetic lenses form a beam that is then scanned over the treated surface. Important parameters for ion implantation are the types of implanted ions, beam energy, flux, and current density [5,7,11,12].

In the work of Nakai et al. [18], it was shown that when high-temperature oxidation in nitrogen gas is used for surface treatment of β-alloys, which takes place at temperatures of 750–950 °C, the α-phase in the nitriding region is stabilized on the surface. On the contrary, nitrogen ion implantation is a low-temperature process that is held at temperatures of 50–500 °C, so in the case of β-alloys, it is a more suitable method. Titanium has a transformation temperature of 882.5 °C when it transforms from a low-temperature allotropic modification with the hexagonal close-packed (HCP) structure to a body-centered (BCC). If lower temperatures are used, there is no damage to the BCC structure of the β-alloys [1,2,3].

There is only a sparse body of literature on nitrogen-implanted β-Ti alloys, mainly the effect of this treatment on the corrosion resistance of these alloys. The effect of nitrides of individual alloying elements on the corrosion resistance of alloys has not been reported anywhere in the literature. One of the first studies that examined the properties of nitrogen-treated β-alloys was conducted by Gordin et al. [12] on a Ti-25Nb-25Ta alloy on which nitrogen was implanted using an energy of 100 keV and a fluence of 5.10^17^ cm^−2^. In this study, it was shown that the implantation of nitrogen ions on this alloy vigorously modified the properties of the surface, as titanium nitrides were formed on it. The nitrogen-treated surface showed almost twice the hardness of the original alloy. Furthermore, it was shown that surface treatment reduced the coefficient of friction and thus prolonged the life of the material. In a ball-on-disk tribological test, they demonstrated a reduction in the coefficient of friction by more than a factor of two compared to the untreated surface. The abrasion footprint was also approximately 85% narrower in the N-implanted specimen. Among other things, electrochemical tests in the simulated body fluid determined a lower corrosion rate compared to that of the native surface. In vitro tests performed on human osteoblasts showed good cytocompatibility of the native sample and the treated surface.

In another work, Vlčák et al. [4] investigated the corrosion properties of the β-alloy Ti-35Ta-7Zr-5Ta. Ion implantation was performed at a voltage of 90 kV and fluences of 1, 2, 4, 6, and 9.10^17^ cm^−2^, and a temperature of 90 °C. The result was that the highest concentration of nitride and the best properties were achieved at a fluence of 4.10^17^ cm^−2^. At the same time, it was found that corrosion resistance is closely related to the concentration of nitrides on the surface.

This work focused on the investigation of the electrochemical properties of β-alloys Ti-36Nb-6Ta, Ti-39Nb, and Ti-36Nb-4Zr, whose surface was modified by nitrogen ion implantation. The same alloys with a native passive layer were used for comparison. To investigate the effect of the nitrides of each element on the corrosion resistance of the alloys, pure metals were selected and modified by the same procedures as the alloys, as their influence has not yet been investigated.

## 2. Results and Discussion

### 2.1. Surface Analysis

The results of the surface analysis are shown in Table 1. It is evident that there is an increase in the concentration of the alloying elements in the surface layer for both alloys, treated or not, compared to bulk. This phenomenon was detected on other beta alloys [4,19,20] and is explained using the Gibbs free energy of the formation of metal oxides [21].

**Figure 1:**
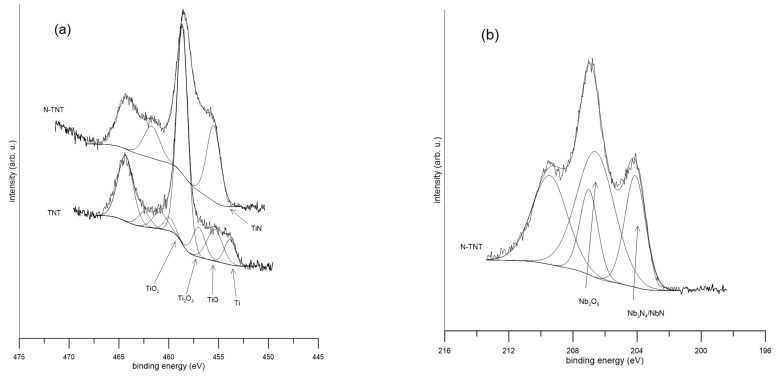

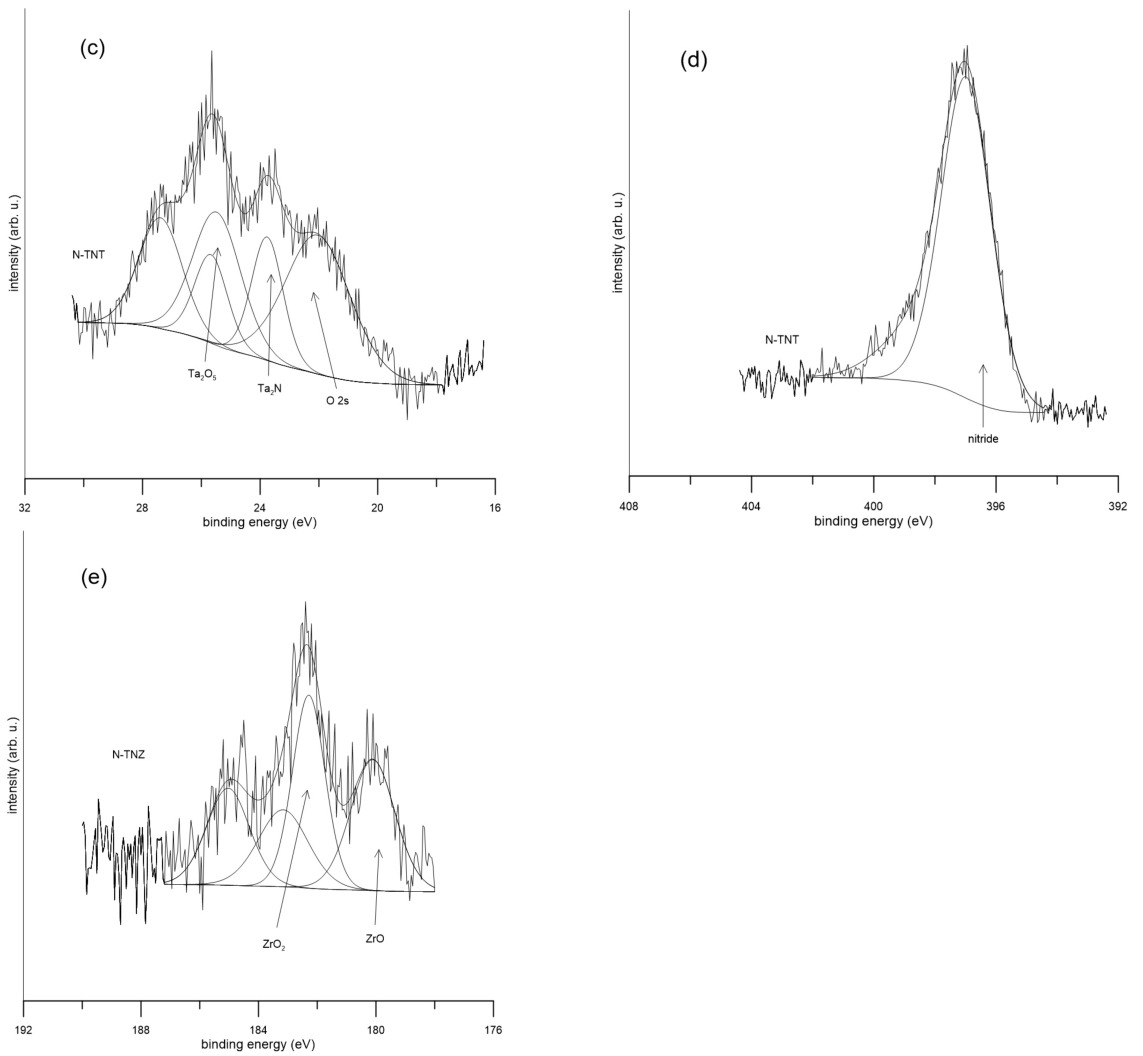
Detailed XPS spectra of selected elements: (**a**) Ti 2p, (**b**) Nb 3d, (**c**) Ta 4f, (**d**) N 1s, (**e**) Zr 3d.

Examples of the XPS spectra of the alloys are shown in Figure 1. It was determined that the surface layer was composed of oxides (TiO_2_, Nb_2_O_5_, Ta_2_O_5_, and ZrO_2_) and nitrides of the alloying elements. In all spectra, except for the detailed spectra of zirconium, a peak belonging to nitrides was detected. Its intensity corresponds to the nitrogen concentration on the surface. After ion implantation, a peak at a binding energy of 455.5 ± 0.3 eV is visible in the titanium spectra, which corresponds to TiN (Figure 1a). The binding energy of 204 ± 0.5 eV was assigned to niobium nitride (Figure 1b), and the binding energy of 24 ± 0.4 eV was assigned to tantalum nitride (Figure 1c). On the surface of both samples of Ti-36Nb-6Ta (treated and reference), the overlap of the O 2s peak in the Ta spectra from oxides of titanium, niobium, and tantalum was detected. There was a peak at 22.1 eV in the Ta 4f spectrum of untreated Ti-36Nb-6Ta that referred to the metal state (Figure 1c). In the case of TNZ, a peak belonging to zirconium nitride was not detected. On the treated Zr sample, only zirconium oxides were detected. The zirconium (Zr 3d 5/2) nitride peak should be detected at an energy of 180.9 eV [17]. In numerous works [22,23,24,25,26,27], zirconium alloys were treated by nitrogen ion implantation at higher temperatures than in our work, usually between 300 °C and 800 °C, and had successfully detected zirconium nitride on their samples. It seems that a lower temperature is not sufficient for ZrN synthesis.

More detailed surface composition analysis of all the samples is shown in Table 2. The results show that after surface treatment, both metals and alloys undergo nitride formation and the elimination of less stable oxides. The formation of stable oxides on the surface should maintain the positive corrosion resistance results of the samples. A positive effect on corrosion resistance is seen when tantalum is used as an alloying element. Many works [28,29,30,31] have shown an increase in the corrosion resistance of Ti alloys when Ta was added. Tantalum forms a stable Ta_2_O_5_ passive film that strengthens the TiO_2_ passive film. In the work of Metikos-Huković [32], the positive effect of niobium on the corrosion resistance of titanium in Hank’s Balanced Salt solution was confirmed. The corrosion stability improved due to the disappearance of anion vacancies in the titanium dioxide lattice. The niobium ions cause a decrease in the concentration of anion vacancies, which are generated by the lower Ti oxidation states. This corresponds with our electrochemical measurements and XPS spectra. The positive effect of Nb on the corrosion resistance of Ti was also confirmed in other studies [33,34].

A similar positive effect is achieved by alloying with zirconium [35,36]. As mentioned earlier, ZrN was not detected on the surface, but for pure Zr, a layer of stable ZrO_2_ oxide was formed by the surface treatment. In the case of TNZ alloy, ZrO and ZrO_2_ oxides were detected. It was further found that almost the same amount of TiN was present on all the alloys.

### 2.2. Electrochemical Behavior

The electrochemical behavior of all samples was studied in a physiological solution, which is the simplest model of the human body environment. The benefit of using this solution is that there are no side-effect processes, such as hydroxyapatite precipitation; therefore, the corrosion behavior could be easily compared.

In Table 3 are the values of polarization resistance and open circuit potential after 12-h exposure. From the results, it can be concluded that after surface modification, the polarization resistance of Nb, Zr, and TNT alloy increased. This increase is probably due to the formation of titanium and niobium nitrides during treatment at the expense of less stable metal oxides. This could be seen from the XPS results in Table 2. The Ta samples and the TN and TNZ alloys showed a decrease in polarization resistance, but still showed good corrosion resistance [20,37,38,39]. N-TNT showed the highest corrosion resistance of the treated alloys determined from the polarization resistance values at the end of 12 h. This suggests the formation of a more resistant surface layer on the modified sample compared to the original alloy. As can be seen from Table 3., ion implantation does not have a significant effect on the corrosion properties of the passive layer of titanium. It is the combination of TiO_2_ and Ta_2_O_5_ in the TNT alloy and subsequent nitrogen treatment that produces a highly corrosion-resistant layer. Similar results were obtained in the work of Gordin et al. [12] when Ti-25Ta-25b alloy showed better electrochemical properties, including polarization resistance, after N implantation. The change in corrosion current density and polarization resistance could also be caused by the change in real area compared to geometric area. As shown in previous work [4], an increase in real area occurs during implantation.

**Figure 2:**
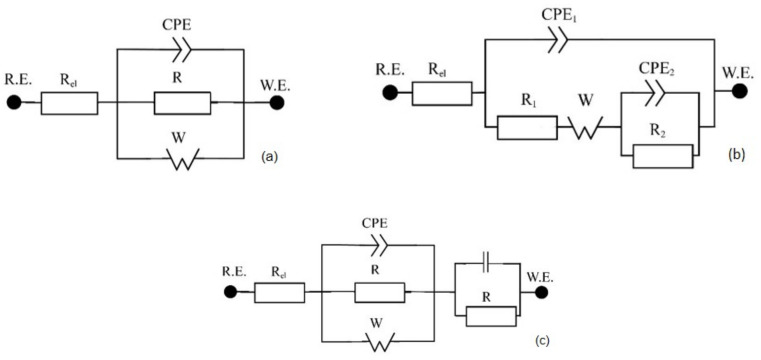
The equivalent circuits used for the evaluation of impedance spectra. Circuit (**a**) was used for samples Ti, Nb, N-Nb, Ta, N-Ta, Zr, TN, N-TN, TNT, N-TNT, TNZ, N-TNZ; circuit (**b**) was used for N-Ti, (**c**) was used for N-Zr.

For a better description of corrosion behavior, electrochemical impedance spectroscopy (EIS) was used. For the evaluation of the EIS spectra, the equivalent circuits shown in Figure 2. were used. Such circuits are used to describe the material–passive layer–electrolyte interface and include the existence of pores on the surface [4,21,40,41,42].

The model in Figure 2a consists of the electrolyte resistance (R_el_), the constant phase element (CPE_1_) with the coefficient α_1_ and infinite Warburg impedance (W). The CPE considers the non-ideal behavior of the system and is defined as Z = [C(jω)^α^] ^−1^, where α takes values from 0 to 1. If α is close to 1, the CPE behaves as a pure capacitor, if it is close to 0, it acts as a pure resistor [40,43,44]. Capacitance C_1_ was calculated using the Equation (1) [41]:

$$C_{1}={\left({\mathrm{CPE}}_{1}\cdot R_{1}\right)}^{\left(1/\propto \right)}/R_{1},$$

The model in Figure 2b additionally contains resistor R_2_ and capacitance C_2_ corresponding to the capacitance of the internal interface. The model in Figure 2c contains R_3_ and capacitance C_3_ in addition to 2a circuit.

**Figure 3:**
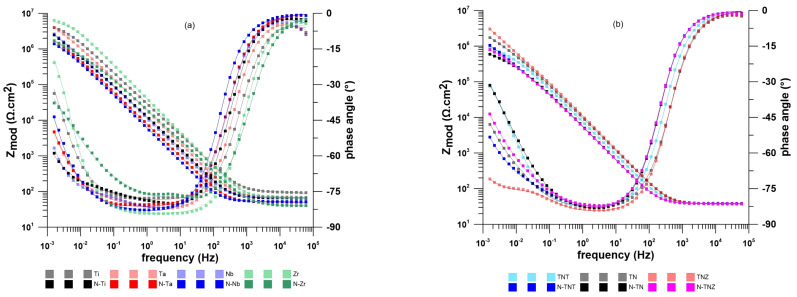
Impedance spectra after 12 h exposure of (**a**) elements and (**b**) alloys in physiological solution.

Figure 3 shows EIS spectra after 12 h of exposure in physiological solution. The calculated values are summarized in Table 4. The chi-squared (χ^2^) error value was on the order of 10^−4^ or lower in all cases, indicating that the equivalent circuits in Figure 2 were appropriate for fitting the EIS spectra in Figure 3. It is evident that after implantation, all the studied materials still had quite a high charge transfer resistance (R_1_), which is reciprocal to the corrosion rate. Furthermore, there was an increase in the CPE_1_ value for all samples tested after ion implantation of nitrogen. This could be attributed to the higher surface porosity which is consistent with the Warburg coefficient values discussed below [41,44]. In the case of N-TNT, this value even increased. As well as the polarization resistance results, it can be concluded that the presence of Ta_2_O_5_ and TaN in the passive layer has a positive effect on the corrosion resistance of this alloy. In the case of all the other samples, R_1_ decreased after ion implantation, which would indicate a decrease in the thickness of the oxide layer on the surface after treatment [4,40]. The W shows the presence of pores and diffusion within them in the passive layer [4,42]. The results show that the Warburg coefficient increased in all samples after implantation. This corresponds to the formation of larger pores in which diffusion is easier [45]. In the case of N-Zr, the value of C_3_ indicates that the pure metal was in a contact with the physiological solution. This is probably due to the formation of an inhomogeneous surface after implantation. It is likely that blistering and exposure of the clean metal occurred during preparation. This created an additional interface which was subsequently detected during exposure.

Considering the results from the XPS analysis, we can assume that the corrosion resistance depends on the formation of titanium and tantalum nitrides. A passive layer consisting of oxides and nitrides effectively prevented chloride and oxygen from coming into contact with the alloy. Wang et al. [46] in their work came to the same conclusion that there is a positive effect of titanium nitrides on corrosion behavior. In their work, they studied the corrosion resistance of Ti, TiN, TiO_2_, and N-TiO_2_ on AISI 316L stainless steel using the EIS in Hank’s solution. It was proved that the fabrication of a surface layer made of Ti and N has a positive effect on the bulk material. Similar results were obtained in the work of Huang et al. [47] using Ti-6Al-7Nb that was nitrided by plasma immersion ion implantation. Corrosion resistance was tested in simulated body fluid (SBF) at 37 °C. The treatment enhanced the corrosion resistance and after implanting screw samples in the femur of adult mini pigs, there was an enhancement in initial osseointegration after implantation for two weeks. Such results correspond with ours.

The use of niobium nitrides [48,49] or tantalum nitrides [50,51] has proven to be a good way to increase corrosion resistance not only in a simulated body environment. Our results are in agreement with these, with a noticeable improvement in corrosion resistance for the N-TNT alloy. In their work, Hussein et al. [52] demonstrated an improvement in the corrosion properties of Ti-20Nb-13Zr at. % alloy in artificial saliva after laser gas nitriding. They attribute this improvement to the formation of TiN on the surface, which led to large capacity, higher charge transfer resistance, and lower corrosion current density compared to the reference sample.

**Figure 4:**
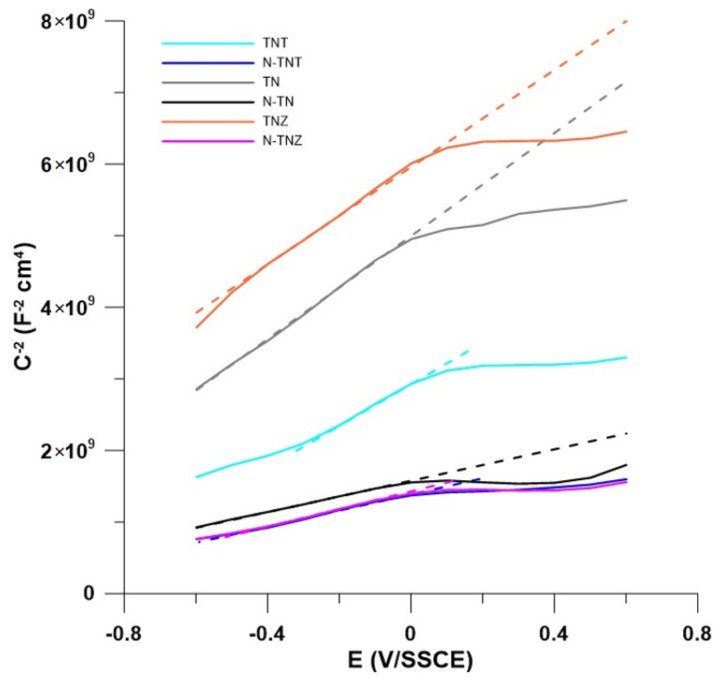
Mott–Schottky plots of alloys in physiological solution.

Figure 4 demonstrates Mott–Schottky plots of the treated and untreated alloys. All samples are n-type semiconductors, since the slope of the linear part is positive throughout, which is consistent with the behavior of TiO_2_. In n-type semiconductors, vacancies behave as electron donors [53,54]. The type of the semiconductor did not change after the ion implantation. However, after the surface treatment of the alloys, all of them showed a decrease in the slope and thus an increase in the number of free charge carriers. All the alloys showed non-uniformity in the donor distribution, which is evident from the fact that there is a change in the slope during the Mott–Schottky curves [55]. This would correspond with the fact, that the surface of ion-implanted samples is not smooth, as was shown in the work of Vlcak et al. [4]. The flat-band potential (E_fb_) can be determined graphically from the intersection of the slope with the zero value of the inverse of the squared capacitance (C^−2^). The graphically evaluated values of E_fb_ are shown in Table 5. In the case on TN and TNT alloys, the E_fb_ decreased, whereas the TNZ sample showed an increase in flat-band potential. The shift in flat-band potential in the alloys is in agreement with the shift of the E_OCP_.

## 3. Materials and Methods

### 3.1. Material Preparation and Surface Modification

Samples of titanium alloys Ti-39Nb, Ti-36Nb-6Ta, and Ti-36Nb-4Zr (i.e., samples designated as TN, TNT, TNZ, and samples with modified surface N-TN, N-TNT, N-TNZ) were 15 mm in diameter and 3 mm in height. For comparison, samples of Ti (Ti grade 2), Nb, Ta, and Zr of the same dimensions were used. These discs were ground on one side with a series of abrasive papers up to P4000 grit, then polished with a silica solution to a mirror-like state. After surface polishing, the specimens were cleaned with distilled water, acetone, and degreased in an ultrasonic cleaner in ethanol. Before exposure, control samples were sterilized in an autoclave at 122 °C for 30 min.

Nitrogen ion implantation was performed using the Tecvac 221 (Tecvac, Cambridge, UK) ion implanter, under conditions of an accelerating voltage of 90 kV at fluence of 2.10^17^ cm^−2^. The working pressure during the process was maintained at about 5.10^−3^ Pa. The ion beam density was kept under 1.5 μA.cm^−2^ so that the temperature did not exceed 90 °C.

### 3.2. Surface Analysis

For surface analysis, the ESCAprobe P (Omicron Nanotechnology Ltd., London, UK) with Al (Kα = 1486.7 eV) X-ray source was used. The spectra were measured with an energy step of 0.05 eV and all binding energies were calibrated to a C 1s peak, 285.0 eV. All samples were cleaned with distilled water, ethanol, and acetone prior to measurement. Binding energies were evaluated using the NIST X-ray Photoelectron Spectroscopy (XPS) Database (accessed on 6 September 2022) [56] and the X-ray Photoelectron Spectroscopy Reference Pages (accessed on 6 September 2022) [57].

### 3.3. Electrochemical Measurements

Measurements were performed with a computer-controlled potentiostat Gamry Instruments Reference 600 (GamrySoftware_6.32.4217, Gamry Instruments Inc., Warminster, PA, USA). The Echem Analyst (GamrySoftware_6.32.4217, Gamry Instruments Inc, Warminster, PA, USA) software was used to calculate the measured data. The experiment was carried out in the standard three-electrode setup. The reference system consisted of a saturated silver–silver chloride electrode with 3 mol/L KCl inner electrolyte (SSCE) (Gamry Instruments Inc, Warminster, PA, USA), graphite rods were used as the auxiliary electrodes, the exposed area was 0.18 cm^2^. The physiological saline solution, 9 g/L NaCl (S9888-500G, Sigma-Aldrich, St. Louis, MO, USA), was used for corrosion measurement.

The measurement cycle consisted of open circuit potential monitoring for 12 h. Next was electrochemical impedance spectra (EIS) (EDC = EOC, E_AC_ = 10 mV rms, frequency range 60 kHz–2 mHz). EIS was followed by the polarization resistance (±20 mV/E_ocp_, 0.125 mV/s). Subsequently, Mott–Schottky curves (from −0.6/E_ocp_ to 0.6/E_ocp_, 0.1 V step, frequency 10 Hz, 10 mV rms amplitude) were measured. All experiments were realized at room temperature.

## 4. Conclusions

1. All β-alloys, Ti-36Nb, Ti-36Nb-6Ta, and Ti-36Nb-4Zr, were successfully nitrided by nitrogen ion implantation with fluence 2.10^17^ cm^−2^.
2. XPS analysis showed the presence of nitrides on all the alloys and pure metals apart from zirconium, more precisely nitrides of titanium, niobium, and tantalum.
3. The corrosion behavior of the modified samples was affected by the alloy composition. Better corrosion resistance was exhibited on the treated Ti-36Nb-6Ta rather than on Ti-39Nb and Ti-36Nb-4Zr. This was probably caused by the presence of tantalum in the Ti-36Nb-6Ta alloy. Tantalum formed oxides and a small percentage of nitrides on the surface, making this alloy more resistant to corrosion in comparison with Ti-39Nb alloy.

## Figures and Tables

**Table 1:** Surface composition of native and modified titanium alloy, determined by XPS (weight %).

	Ti	Nb	Ta	Zr	O	N
TN	31.1	32.9	-	-	36.0	-
N-TN	26.7	42.0	-	-	22.6	8.7
TNT	35.2	27.8	10.5	-	26.5	-
N-TNT	26.2	38.2	7.0	-	19.9	8.7
TNZ	32.3	32.7	-	1.5	33.5	-
N-TNZ	26.5	38.2	-	3.5	21.3	10.5

**Table 2:** Surface composition analysis of the samples, determined by XPS (atomic %).

	Ti	N-Ti	Nb	N-Nb	Ta	N-Ta	Zr	N-Zr	TN	N-TN	TNT	N-TNT	TNZ	N-TNZ
TiO	7.9	-	-	-	-	-	-	-	5.2	-	14.2	-	9	-
TiO_2_	75.6	57.2	-	-	-	-	-	-	86.3	55.6	66.8	54.4	41.3	56.5
Ti_2_O_3_	11.9	28.7	-	-	-	-	-	-	8.5	17.2	10.8	20	8.4	19.8
Ti_x_O_x_	4.6	-	-	-	-	-	-	-	-	-	8.2	-	5.7	-
TiN	-	14.1	-	-	-	-	-	-	-	27.2	-	25.6	-	23.7
Nb	-	-	17.1	-	-	-	-	-	5.1	-	5.9	-	11.3	-
NbO	-	-	15.1	-	-	-	-	-	5.1	-	18.1	-	18.1	-
NbO_2_	-	-	11.8	-	-	-	-	-	-	-	19.4	-	16.9	-
Nb_2_O_5_	-	-	56	60.3	-	-	-	-	87.7	58.8	56.6	67.1	53.7	55.8
NbN	-	-	-	39.7	-	-	-	-	-	41.2	-	32.9	-	44.2
Ta	-	-	-	-	-	-	-	-	-	-	26	-	-	-
Ta_2_O_5_	-	-	-	-	100	59.2	-	-	-	-	66.7	41.3	-	-
TaN	-	-	-	-	-	40.8	-	-	-	-	-	25.1	-	-
Zr	-	-	-	-	-	-	12	-	-	-	-	-	-	-
ZrO_2_	-	-	-	-	-	-	88	100	-	-	-	-	100	60.3
ZrO	-	-	-	-	-	-	-	-	-	-	-	-	-	39.7

**Table 3:** Corrosion parameters determined from electrochemical measurements in physiological solution.

Material	R (Ω·cm^2^)		E_ocp_ (V/SSCE)	
	Native	N-	Native	N-
Ti	6.2 × 10^6^	6.2 × 10^6^	0.012	0.201
Nb	9.9 × 10^5^	2.2 × 10^6^	−0.216	0.118
Ta	6.2 × 10^6^	3.1 × 10^6^	0.030	0.104
Zr	6.7 × 10^6^	7.1 × 10^7^	−0.207	0.062
TN	2.8 × 10^6^	7.3 × 10^5^	0.067	0.001
TNT	1.4 × 10^6^	2.3 × 10^6^	0.097	0.063
TNZ	1.6 × 10^7^	1.3 × 10^6^	0.032	0.083

**Table 4:** Values of equivalent circuit used for the EIS spectra, C_1_ was calculated.

	Eq. Circuit	R_el_ (Ω·cm^2^)	CPE_1_ (S.s^α^/cm^2^)	α_1_	C_1_ (F/cm^2^)	R_1_ (Ω·cm^2^)	R_2_ (Ω·cm^2^)	W (S.s^0.5^/cm^2^)	C_2_ (F/cm^2^)	CPE_2_ (S.s^α^/cm^2^)	α_2_	R_3_ (Ω·cm^2^)	C_3_ (F/cm^2^)	Χ^2^
Ti	A	94.4	6.7 × 10^−6^	0.9	1.0 × 10^−5^	6.0 × 10^6^	-	2.2 × 10^−7^	-	-	-	-	-	2.3 × 10^−4^
N-Ti	B	65.2	1.3 × 10^−5^	0.9	3.3 × 10^−5^	-	1.9 × 10^6^	4.3 × 10^−6^	3.9 × 10^−5^	-	-	-	-	6.1 × 10^−5^
Nb	A	67.9	1.6 × 10^−5^	0.9	2.9 × 10^−5^	1.7 × 10^7^	-	1.6 × 10^−6^	-	-	-	-	-	4.3 × 10^−4^
N-Nb	A	52.1	2.9 × 10^−5^	0.9	4.7 × 10^−5^	2.6 × 10^6^	-	1.6 × 10^−6^	-	-	-	-	-	4.4 × 10^−5^
Ta	A	70.1	9.6 × 10^−6^	0.9	1.6 × 10^−5^	1.3 × 10^7^	-	9.5 × 10^−7^	-	-	-	-	-	2.0 × 10^−4^
N-Ta	A	50.1	2.3 × 10^−5^	0.9	3.9 × 10^−5^	4.1 × 10^6^	-	1.3 × 10^−6^	-	-	-	-	-	1.3 × 10^−4^
Zr	A	68.0	4.3 × 10^−6^	0.9	6.2 × 10^−5^	6.9 × 10^6^	-	9.0 × 10^−8^	-	-	-	-	-	1.5 × 10^−4^
N-Zr	C	39.9	9.3 × 10^−6^	0.9	1.4 × 10^−5^	4.3 × 10^6^	-	3.3 × 10^−6^	-	-	-	7.6 × 10^4^	1.0 × 10^−4^	2.3 × 10^−4^
TN	A	37.1	1.7 × 10^−5^	0.9	2.9 × 10^−5^	5.6 × 10^6^	-	2.7 × 10^−6^	-	-	-	-	-	7.4 × 10^−5^
N-TN	A	38.1	2.8 × 10^−5^	0.9	4.0 × 10^−5^	1.1 × 10^6^	-	7.2 × 10^−6^	-	-	-	-	-	1.9 × 10^−4^
TNT	A	39.1	2.2 × 10^−5^	0.9	3.2 × 10^−5^	1.4 × 10^6^	-	3.5 × 10^−6^	-	-	-	-	-	2.9 × 10^−4^
N-TNT	A	38.3	3.1 × 10^−5^	0.9	5.6 × 10^−5^	8.0 × 10^6^	-	5.8 × 10^−6^	-	-	-	-	-	1.0 × 10^−4^
TNZ	A	37.7	1.6 × 10^−5^	0.9	3.2 × 10^−5^	3.1 × 10^7^	5.6 × 10^5^	-	-	3.8 × 10^−6^	0.7	-	-	4.5 × 10^−5^
N-TNZ	A	38.0	3.0 × 10^−5^	0.9	4.8 × 10^−5^	2.6 × 10^6^	-	7.0 × 10^−6^	-	-	-	-	-	6.7 × 10^−5^

**Table 5:** Flat-band potential determined from Mott–Schottky plots.

	E_fb_ (V)	
	Native	N-
TN	−1.249	−1.264
TNT	−0.997	−1.233
TNZ	−1.549	−1.016

## Data Availability

Data is contained within the article.
